# Atlanta Society of Medicine

**Published:** 1889-02

**Authors:** 


### Society Reports.

### ATLANTA MEDICAL SOCIETY.

MEETING JANUARY 3, 1889.

Dr. Hunter P. Cooper in the chair.

Dr. E. V. Joye exhibited forceps, which he named his "Paring and Suture Clamp Forceps," designed by him, for the operation of simple vesico-vaginal fistula, laceration of the perineum, colporrhaphy (anterior and posterior), elytrorrhaphy, etc., and read a paper descriptive of the instrument and the manner of using it, in which he said the instrument consisted of two blades, each eleven inches long, which are crossed and locked similarly to the "Hodge Forceps." Two inches of the extremities of the blades are made round, into which are inserted five hooks equidistant. The hooks are sharp and small at the free end, and thick where they enter the blades (so made as to avoid tearing out easily). Shanks four inches long, containing the screw lock of the "Hodge Forceps." The handles are five inches long, slightly curved downwards, (in order to be out of the way of operator,) with ratchet to hold and fix the blades in any desired position, either extended or brought together. The operation for vesico-vaginal fistula is performed as follows: Patient in position recommended by Dr. J. Marion Sims—chloroform administered. Dr. Sims' duck-bill speculum introduced, an assistant holding up the right side of the nates. The instrument being unlocked, the left hand blade is passed in the vagina, and hooked into the mucous tissue on the right side of the fistula (not too near the edge), and rotated from *left* to *right*, thus bringing the lock up, with the points of the hooks looking towards the *right side*. The handle is held down, and the *right hand blade* is then passed in over the other, and the hooks hooked into the *left side*

of the fistula, and rotated from *right* to *left*, and brought above and across the left hand blade. The blades are locked; the handles brought together and fixed by the ratchet; the fistula is thus firmly held, with its edges everted. The paring is done with Dr. Emmet's straight and curved scissors. Needles, armed with silver wire, are passed, one in front of first hook, and then between each hook, and one on the outside of last hook. Sutures twisted up and cut off. The blades are then unlocked, rotated in the opposite direction from that and hooked into the vaginal tissue. The blades are then withdrawn, and the operation is completed.

The advantages gained are: 1st, by fixing the fistula with the instrument, the everted edges can be pared by one stroke of the scissors; 2d, the blades, constricting the everted edges of fistula, furnish support for the passage of the needle, and the better twisting and adjustment of the sutures; 3d, the instrument gives the operator entire control of the parts; lastly, it renders the operation comparatively easy, and reduces the time of operation two-thirds. He also said that the instrument could be used with advantage in all operations wherein the principles of eversion, elevation and continuous adjustment of tissue were necessary, such as perineorrhaphy, colporrhaphy, elytrorrhaphy, etc.

Dr. Hardon remarked that it was always a difficult matter to pare the edge of a fistula properly, and that he thought the forceps presented by Dr. Joye, had come to fill a long felt want—that the instrument would overcome the difficulty found in paring the edges of fistula, etc.; also that he would certainly procure one and use it.

Dr. Hardon reported a case of Tait's operation, and exhibited the ovaries, with tubes attached, which he had removed. Dr. Hardon said the patient, aged 35 years, mother of children, had suffered for a long time, some eight months, with intense and constant pain in the left inguinal region, which required as much as two grains of morphia to temporarily relieve. She had consulted, and had been treated by, other prominent physicians, before coming to him—for a long period—without any benefit or relief. He used anodynes, the warm douche, and in fact all the internal and external remedies applicable to such a case, without any beneficial effect. The continuance and severity of the pain, and the general impaired condition of the patient, consequent upon such protracted suffering, induced him to propose operation, which was agreed to; accordingly, with the able assistance of Drs. Cooper, Jones, Westmoreland and Johnson, he performed Battey's operation, by the abdominal section. The patient making an excellent recovery. In showing the ovaries and tubes removed, Dr. Hardon said upon the left ovary could be seen a small cyst, and that the organ, at time of removal, contained a hæmatoma as large as a walnut. He called attention to the connection between seat of pain and the diseased ovary, the seat of pain being the left inguinal region. The Doctor said that the right ovary showed, besides, cystic degeneration, and with the consent of the patient he removed it also, because he deemed it prudent to do so, fearing that at some time it might develop the same condition that existed in the left, and bring about a similar degree of distress. Dr. Hardon mentioned in conclusion, that up to the present time, four weeks from date of operation, the patient was well and giving every evidence of permanent relief.

Dr. Todd asked Dr. Hardon if he noticed anything of a cancerous nature about the patient.

Dr. Hardon replied that he did not.

Dr. McRea reported a case of puerperal convulsions. Said he was called in consultation with Dr. C. E. Murphy, found the patient in convulsions, with os uteri about the size of a nickle, gave chloroform, dilated the os with his finger, and discovered the head engaging the brim of pelvis; using antiseptics thoroughly and freely, applied the forceps and delivered very rapidly. After delivering, the patient had no more convulsions, and that the child was perfect and healthy. Dr. McRea added that at no time was the patient's temperature above normal, and that both mother and child were still progressing favorably.

Dr. Hardon said that he was very glad that Dr. McRea had treated the case as he had done—that he believed rapid delivery to be the true and scientific way of treating puerperal convulsions; that he had long held this opinion, and mentioned in connection that Lusk and other authorities gave as their experience, that convulsions would and did cease as soon as the uterus was emptied. He thought that delaying was very dangerous ; that many lives were lost thereby. Dr. Hardon thought forcible dilation and rapid delivery were preferable, in every case, to blood-letting, morphia and waiting.

Dr. Duncan agreed with Dr. Hardon, and mentioned that he had on two or three occasions employed the "forcible dilation and quick delivery treatment," and had been successful in relieving the patients and bring them through all right.

Dr. Divine asked Dr. Hardon if it was not very difficult to dilate the os in some cases.

Dr. Hardon answered that if the woman had been in labor for sometime, that it was not difficult ; that he was in the habit of using Tarnier's narrow blade axis-traction forceps, and would apply them and grasp the head, whether it had entered the brim of the pelvis, or was high up and not engaging, and would complete the dilatation as he brought the head down to and through the os, and that in this way he had never failed to empty the uterus and bring about good results.

